# Intestinal toxicity of deoxynivalenol is limited by supplementation with *Lactobacillus plantarum* JM113 and consequentially altered gut microbiota in broiler chickens

**DOI:** 10.1186/s40104-018-0286-5

**Published:** 2018-10-08

**Authors:** Shengru Wu, Yanli Liu, Yongle Duan, Fangyuan Wang, Fangshen Guo, Fang Yan, Xiaojun Yang, Xin Yang

**Affiliations:** 1 College of Animal Science and Technology, Northwest A&F University, Yangling, Shaanxi China; 2 College of animal science and Technology, Hebei Agricultural University, Baoding, Hebei China

**Keywords:** Broiler chickens, Deoxynivalenol, Gut microbiota, *Lactobacillus plantarum* JM113, mRNA sequencing, 16S rRNA gene sequencing

## Abstract

**Background:**

Limited research has focused on the effect of *Lactobacillus* on the intestinal toxicity of deoxynivalenol (DON). The present study was conducted to investigate the role of *Lactobacillus plantarum (L. plantarum)* JM113 in protecting against the intestinal toxicity caused by DON.

**Methods:**

A total of 144 one-day-old healthy Arbor Acres broilers were randomly distributed into 3 treatments, including the CON (basal diet), the DON (extra 10 mg/kg deoxynivalenol), and the DL (extra 1 × 10^9^ CFU/ kg *L. plantarum* JM113 based on DON group) treatments. The growth performance, organ indexes, intestinal morphology, pancreatic digestive enzymes, intestinal secreted immunoglobulin A (sIgA), jejunal transcriptome, and intestinal microbiota were evaluated.

**Results:**

Compared with the CON and DL groups, the DON supplementation altered intestinal morphology, especially in duodenum and jejunum, where villi were shorter and crypts were deeper (*P* < 0.05). Meanwhile, the significantly decreased mRNA expression of jejunal claudin-1 and occludin (*P* < 0.05), ileal *rBAT* and jejunal *GLUT1* of 21-day-old broilers (*P* < 0.05), as well as duodenal *PepT1* and ileal *rBAT* of 42-day-old broilers were identified in the DON group. Moreover, supplementation with *L. plantarum* JM113 could increase duodenal expression of *IL-10* and *IL-12* of 21-day-old broilers, ileal sIgA of 42-day-old broilers, and the bursa of Fabricius index of 21-day-old broilers. Further jejunal transcriptome proved that the genes related to the intestinal absorption and metabolism were significantly reduced in the DON group but a significant increase when supplemented with extra *L. plantarum* JM113. Furthermore, the bacteria related to nutrient utilization, including the Proteobacteria, *Escherichia*, *Cc-115 (P* < 0.05*)*, *Lactobacillus* and *Prevotella* (*P* < 0.1) were all decreased in the DON group. By contrast, supplementation with *L. plantarum* JM113 increased the relative abundance of beneficial bacterium, including the Bacteroidetes, *Roseburia*, *Anaerofustis*, *Anaerostipe*, and *Ruminococcus bromii* (*P* < 0.05). Specifically, the increased abundance of bacteria in the DL group could be proved by the significantly increased caecal content of propionic acid, n-Butyric acid, and total short-chain fatty acid.

**Conclusions:**

*L. plantarum* JM113 enhanced the digestion, absorption, and metabolic functions of the gut when challenged with DON by reducing the injury to intestinal barriers and by increasing the abundance of beneficial bacterium.

**Electronic supplementary material:**

The online version of this article (10.1186/s40104-018-0286-5) contains supplementary material, which is available to authorized users.

## Introduction

The trichothecene mycotoxin deoxynivalenol (DON) is commonly found in feedstuff following infestation by the fungus *Fusarium* [1, 2]. Intestinal epithelial cells are exposed to DON following the ingestion of contaminated diets [3]. Exposure to DON can induce excessive production of free radicals [4], inhibit protein synthesis [5], and activate critical cellular kinases involved in signal transduction related to proliferation, differentiation, and apoptosis [6], which could further induce cytotoxicity and apoptosis, induce gastrointestinal inflammation and necrosis, and disturb the gut barrier [7]. In contrast to the acute effect of DON in humans, piglets, and other mammals, which results in diarrhoea, vomiting, leukocytosis, gastrointestinal haemorrhage and ultimately death [8, 9], the relatively high tolerance of poultry to DON has been attributed to its low bioavailability and effective metabolism [10]. Whereas sub-chronic symptoms including decreased body weight and feed consumption, as well as damage to the intestinal barrier, were also widely identified and worth further study [11].

There are extensive functional interactions between the gut microbiota and host metabolism or immunity [12, 13]. The immune, metabolic, and digestive systems as well as the diet and stress during animal feeding, are all involved in modulating the gut microbiota [14–16]. Recently, it was reported that microbiota is key target for dietary mycotoxins, in which DON was the most documented [17, 18]. The altered microbiota, which in response to feed-derived DON, could contribute to the absorption and utilization of nutrients as well as play a significant part in enhancing body immunity [19]. Herein, we studied the roles of altered microbiota and the alterations in host gene regulation in response to exposure to DON.

A number of physical or chemical detoxification systems have been reported to control the adverse effects of mycotoxins in animal production systems [20, 21]. However, the structure of DON prevents the absolute removal or adsorption of DON. In comparison, *Lactobacillus* is considered to be a good candidate since it can improve intestinal health by stimulating the immune system, modulating gut microbiota, and preventing oxidative damage [22], thereby eliminating the hazards of DON. The *Lactobacillus plantarum* (*L. plantarum*) JM113 strain has high antioxidant activity and thereby supplementation in feed has the potential to protect the integrity of the intestinal barrier in broilers challenged with DON [22]. This study was carried out to evaluate the beneficial effects of *L. plantarum* JM113 on the intestinal toxicity of DON in broilers. By using the Illumina high-throughput sequencing technique, the altered microbiota and alterations in gene regulation in response to DON challenge as well as the protective effect of *L. plantarum* JM113 on intestinal function were studied.

## Materials and methods

### Toxins and bacterial strains

According to the procedures described by Yang et al. [22], DON was manufactured by inoculating rice with *Fusarium graminearum* ACCC 37687 and then was prepared in powder. The contaminated diets were prepared by adding the powder of DON into the feed directly.

*L. plantarum* JM113 was isolated from the intestines of a healthy Chinese Tibetan chicken according to Brisbin et al. [23]. The bacteria was identified by sequencing the V3 region of the 16S rRNA gene and compared with the nonredundant nucleotides in the GenBank database using BLAST, as described in our previous study [24]. Then, *L. plantarum* JM113 strain was cultured in DeMan, Ragosa and Sharpe (MRS) broth at 37 °C for 24 h. After incubation, culture was centrifuged at 6,000×*g* for 5 min at 4 °C, after which the pellets were washed with distilled water by centrifuging at 8,000×*g* for 10 min. The viability of the freeze-dried bacteria was 5 × 10^10^ CFU/g and was stored at − 80 °C until further use.

### Birds and experimental design

All the birds and experimental protocols in this study were approved by the Institution Animal Care and Use Committee of the Northwest A&F University (protocol number NWAFAC1008). Based on a single factor experimental design, a total of 144 one-day-old Arbor Acres male broilers (47.89 ± 1.17 g) were randomly assigned to 3 groups with 6 replications and 8 birds per replicate, including 3 treatments: the control (CON) group was supplied with a corn-soybean basal diet, the deoxynivalenol (DON) group was supplied with extra 10 mg/kg deoxynivalenol, and the deoxynivalenol + *L. plantarum* JM113 (DL) group was supplied with 1 × 10^9^ CFU/ kg *L. plantarum* JM113 when challenged with 10 mg/kg deoxynivalenol. The basal diet was typical of diets commonly used in the Northwestern District of China to meet the National Research Council (NRC, 1994) recommendations (Additional file 1: Table S1). All birds had ad libitum access to feed and water. In brief, by using a high performance liquid chromatography (HPLC) technique following the method of Yang et al. [22], the dietary concentration of DON was 0.489 ± 0.085 mg/kg in the starter control feed (1–21 d of age) and 0.456 ± 0.080 mg/kg in the grower control feed (22–42 d of age). Meanwhile, in the DON-contaminated starter and grower feeds, the concentrations of DON were 10.268 ± 0.090 mg/kg and 10.764 ± 0.090 mg/kg, respectively. Moreover, by using plate counting method, the viability of probiotic was greater than 9 × 10^8^ CFU/kg in feed of the DL group and was not detected in feed of the control group.

The experiment lasted for 42 d. On d 21 and 42, broilers were weighed, and the feed consumptions were recorded by replication. Average daily weight gain, average daily feed intake, and the ratio of feed to gain were calculated.

### Sample collection and determination of organ index

The samples were respectively gathered at 21 and 42 d of age. For each sampling, one bird from each replicate was randomly selected and weighed after fasting for 12 h. The blood samples were collected and the plasma samples were prepared, followed by euthanasia by exsanguination after intravenous administration of 3% sodium pentobarbital (25 mg/kg body weight; Sigma, USA) and immediately dissected. All efforts were made to minimize the animals’ suffering. Firstly, immune organs (liver, thymus, spleen, and bursa) were collected and weighed immediately. Organ indexes were expressed relative to body weight (g of organ/kg of body weight). Then, by removing the contamination of intestinal contents, the middle complete duodenal, jejunal, and ileal segments with lengths of 3 cm were collected and fixed in 10% buffered formalin for at least 48 h for further histological processing. Next, the duodenal, jejunal, ileal, and caecal content samples; the duodenal, jejunal, and ileal mucosa samples; and the whole pancreas samples were collected into 2 mL Eppendorf tubes and frozen immediately in liquid nitrogen. The duodenal, jejunal, and ileal segments were stored at 4 °C and the other samples were stored at − 80 °C until analyses.

### Determination of intestinal morphology

The middle complete duodenal, jejunal, and ileal segments which were fixed in 10% buffered formalin, were used for analysis of intestinal morphology. After fixation, fixed samples were dehydrated and cleared. Then, intestinal samples were cut and inserted into cassettes, which were embedded in liquid paraffin. Next, 5 μm paraffin sections were cut using the microtome and stained with haematoxylin-eosin. Villus height and crypt depth were determined using a phase contrast microscope [25].

### Measurement of the activities of pancreatic digestive enzymes and small intestinal secretory immunoglobulin A (sIgA)

The activities of pancreatic digestive enzymes, including the amylase, chymotrypsin, and lipase, were measured by spectrophotometric methods according to the manufacturer’s instructions (Jiancheng Biological Engineering Research Institute, Nanjing, China). Moreover, the sIgA content of mucous membrane samples of the duodenum, jejunum, and ileum were measured by enzyme linked immunosorbent assay (ELISA) kits (YuanMu Biological Technology Co. Ltd., Shanghai, China) for chickens.

### Microbial DNA extraction, 16S rRNA gene amplification of the V3 + V4 region, sequencing, and bioinformatics analysis

A total of 18 caecal content samples from 42-day-old broilers of 3 different treatments were used for DNA extraction using QIAamp DNA Stool Mini Kit (Qiagen, Germany). The quantity and quality of those 18 DNA samples were further assessed by Nanodrop ND-1000 spectrophotometer (Thermo Scientific, USA) and then stored at − 80 °C until sequencing analysis.

16S rRNA gene amplicons were used to determine the diversity and structural comparisons of the bacterial species in each of these samples using Illumina MiSeq sequencing at Novogene Bioinformatics Technology Co., Ltd., Beijing, China. The PCR amplifications were conducted with the barcoded primer pair that amplifies the V3 + V4 fragments of the 16S rRNA gene [26]. Pyro-sequencing was conducted on an Illumina HiSeq PE250 platform.

Paired-end reads were assigned to each sample based on their unique barcode and truncated by cutting off the barcode and primer sequence. After initial trimming, the merged reads were obtained with FLASH v1.2.7 (http://ccb.jhu.edu/software/FLASH/) based on overlapping regions within the paired-end reads [27], and the splicing sequences were called raw tags. Quality filtering on the raw tags was performed according to the QIIME (V1.7.0, http://qiime.org/index.html) quality control process [28], and all sequences shorter than 2,000 bp in length and having a quality score lower than 25 in the raw reads were removed. The chimaera sequences were also removed through the UCHIME algorithm (http://www.drive5.com/usearch/manual/uchime_algo.html) and the Gold database (http://drive5.com/uchime/uchime_download.html) [29, 30].

Finally, the high-quality clean tags were obtained. These sequences were classified into the same operational taxonomic units (OTUs) at an identity threshold of 97% similarity using UPARSE software (UPARSE v7.0.1001, http://drive5.com/uparse/) [31]. For each OTU, a representative sequence was screened and used to assign taxonomic composition using the RDP Classifier (Version 2.2, http://sourceforge.net/projects/rdp-classifier/) and the Green Gene database (http://greengenes.lbl.gov/cgi-bin/nph-index.cgi) [32, 33]. The taxon abundance of each sample was separated into the phylum, class, order, family, and genera levels. The MetaStat was used to identify the effect of DON on gut microbiota and the effect of *L. plantarum* JM113 on gut microbiota when challenge with DON.

### RNA isolation of jejunal mucous membrane, sequencing, and bioinformatics analysis

Total RNA from 9 jejunal mucous membrane samples (from 3 replications) from 42-day-old broilers were extracted using the TRIzol reagent (Invitrogen, CA, USA). Specifically, DNaseI was used during the RNA isolation process to avoid contamination with genomic DNA. The quantity and purity of total RNA were analyzed by a NanoDrop® ND-1000 spectrophotometer (Thermo Scientific, MA, USA), and the integrity of RNA was assessed with the Bioanalyzer 2100 and RNA Nano6000 LabChip Kit (Agilent, CA, USA). Only samples that had an OD260/280 > 1.8, OD260/230 > 2.0, and a RNA integrity number > 7.0 were used for further sequencing.

Approximately 3 μg of total RNA from each sample was used to prepare an mRNA library according to the Illumina® TruSeq™ RNA sample preparation protocol. Then, the paired-end sequencing (2 × 125 bp) was performed on an Illumina Hiseq 2500 at the Novogene Bioinformatics Institute (Beijing, China). Raw data were obtained using CASAVA v1.8+ (Illumina). The index of the reference genome was built using Bowtie v2.2.3 [34] and sequences were aligned to the chicken genome (*Gallus gallus* 5, Ensembl 85; http://www.ensembl.org/Gallus_gallus/Info/Index) using TopHat [35]. Sequence segments were spliced and annotated, and transcript expressions were calculated by Cufflinks [36]. Fragments per kilobase of exon per million mapped reads (FPKM) was employed to quantify the gene expression [36]. Based on negative binomial distribution, differentially expressed genes (DEGs) were screened out by using DESeq with an adjusted *P* < 0.05 and fold change > 2 or < 0.5. Gene Ontology (GO) enrichment analysis for the screened DEGs was carried out using the GOseq platform [37]. Kyoto Encyclopedia of Genes and Genomes (KEGG) pathway enrichment analysis for the DEGs was performed by using KOBAS software [38]. *P* < 0.05 was defined as significantly enriched GO terms or KEGG pathways.

### Quantitative real-time PCR (qRT-PCR) analysis

Approximately 1 μg of total RNA was reverse transcribed using the PrimeScript™ RT reagent Kit with gDNA eraser (TaKaRa, Dalian, China). qRT-PCR was performed using SYBR® Green PCR Master Mix (TaKaRa, Dalian, China). A 20 μL PCR mixture was quickly prepared. Primers for *β-actin* (internal control genes) and tested mRNAs were designed using Primer-BLAST (http://www.ncbi.nlm.nih.gov/tools/primer-blast/) and listed in Additional file 1: Table S2. Briefly, the tested mRNAs were included four part: 1) genes which were involved in nutrient transport: *PepT1*, *rBAT*, *SGLT1*, *GLUT1*, and *y*^+^*LAT2*; 2) genes of intestinal tight junction components: *Claudin-1*, *occludin*, and *ZO1*; 3) genes of cytokines: *TNF-α*, *IFN-γ*, *IL-10*, and *IL-12*; 4) DEGs selected by RNA sequencing: *MST1*, *CFTR*, *Fgfl19*, *PFKFB3*, and *HBG2*. The PCR was conducted in an iCycler iQ5 multicolour real-time PCR detection system (Bio-Rad Laboratories) and programmed as follows: 95 °C for 10 min; 40 cycles of 95 °C for 10 s; 60 °C for 30 s; 72 °C for 30 s; and 72 °C for 5 min. All samples were examined in triplicate. All data were analyzed using the 2^−ΔΔCt^ method [39].

### Determination of volatile fatty acid (VFA) in caecal digesta

Caecal VFA concentrations were determined via gas chromatography (Agilent 7890A, Agilent Technologies, Santa Clara, CA) according to the procedures described by Shen et al. [40].

### Statistical analysis

The analysis was done by using One-way ANOVA using SPSS 21.0 software with replicates as experiment units and differences were considered to be statistically significant at *P* < 0.05. Significant differences at the 0.05 level due to treatments were separated by Duncan’s multiple range tests.

## Results

### *L. plantarum* JM113 reduced the intestinal histological damage and injury to intestinal barriers induced by DON

In 21-day-old broilers, compared with the control group, a significant decrease in duodenal villus height (*P* < 0.001), increase in duodenal crypt depth (*P* = 0.012), as well as a decrease in the duodenal ratio of villus height to crypt depth (V:C) (*P* = 0.001) was detected in the DON group (Table 1). Conversely, chickens exposed to *L. plantarum* JM113 along with DON showed normal villus length, crypt depth, and normal V:C value of the duodenum when compared with the control group, which showed a significantly increased villus length, decreased crypt depth and an increased duodenal V:C value in the DL group (*P* < 0.05), when compared with the DON group (Table 1). In 42-day-old broilers, compared with the control group, the significantly decreased duodenal and jejunal villus height (*P* < 0.001 and *P* = 0.012) and a significantly decreased jejunal V:C (*P* = 0.020) were detected in the DON group (Table 1). The DL group also showed normal villus length and normal V:C value when compared with the control group, which also had significantly increased villus length in the duodenum and jejunum as well as increased V:C value of the jejunum in the DL group (*P* < 0.05) when compared with the DON group (Table 1).

**Table 1:** Effects of *L. plantarum* JM113 and DON on intestinal morphology and organ index of broiler chickens

Item			Treatment^1^			SEM	*P*-value
			CON	DON	DL		
21-day-old broilers	Duodenum	Villus height, μm	670.77^b^	638.22^c^	710.33^a^	7.21	< 0.001
		Crypt depth, μm	82.61^b^	94.5^a^	89.33^ab^	1.67	0.012
		Villus height: Crypt depth	8.23^a^	6.84^b^	8.12^a^	0.17	0.001
	Jejunum	Villus height, μm	478.66	484.27	448.33	9.38	0.247
		Crypt depth, μm	74.94	76.88	74.44	1.24	0.706
		Villus height: Crypt depth	6.4	6.49	6.07	0.16	0.549
	Ileum	Villus height, μm	328.44	342.27	312.08	5.50	0.102
		Crypt depth, μm	65.72	66.00	65.75	0.91	0.99
		Villus height: Crypt depth	5.04	5.24	4.77	0.11	0.261
	Liver, g/kg		23.50	24.87	23.87	0.30	0.166
	Pancreas, g/kg		3.98	4.04	3.75	0.14	0.726
	Thymus, g/kg		1.88	1.91	2.28	0.11	0.284
	Spleen, g/kg		0.95	1.30	0.84	0.11	0.207
	Bursa of Fabricius, g/kg		1.93^ab^	1.52^b^	2.56^a^	0.15	0.016
42-day-old broilers	Duodenum	Villus height, μm	779.83^a^	693.00^c^	740.77^b^	9.11	< 0.001
		Crypt depth, μm	115.22	111.78	112.83	1.77	0.726
		Villus height: Crypt depth	6.87	6.31	6.61	0.13	0.223
	Jejunum	Villus height, μm	645.05^a^	586.77^b^	655.83^a^	10.41	0.012
		Crypt depth, μm	95.55	98.44	100.05	1.49	0.468
		Villus height: Crypt depth	6.82^a^	5.97^b^	6.59^a^	0.13	0.020
	Ileum	Villus height, μm	416.22	400.00	433.55	6.71	0.124
		Crypt depth, μm	62.38	64.72	63.89	1.04	0.639
		Villus height: Crypt depth	6.75	6.24	6.86	0.14	0.186
	Liver, g/kg		23.97	24.33	22.41	1.20	0.806
	Pancreas, g/kg		2.21	2.24	2.25	0.06	0.970
	Thymus, g/kg		2.02	2.44	2.10	0.15	0.493
	Spleen, g/kg		2.09	1.94	1.68	0.11	0.389
	Bursa of Fabricius, g/kg		1.16	1.03	1.37	0.11	0.547

^a-c^Mean values within a row have same superscript letters were not significant difference (*P* < 0.05)

^1^CON means the control group, DON means the deoxynivalenol group, DL means the deoxynivalenol+ *L. plantarum* JM113 group

**Fig. 1:**
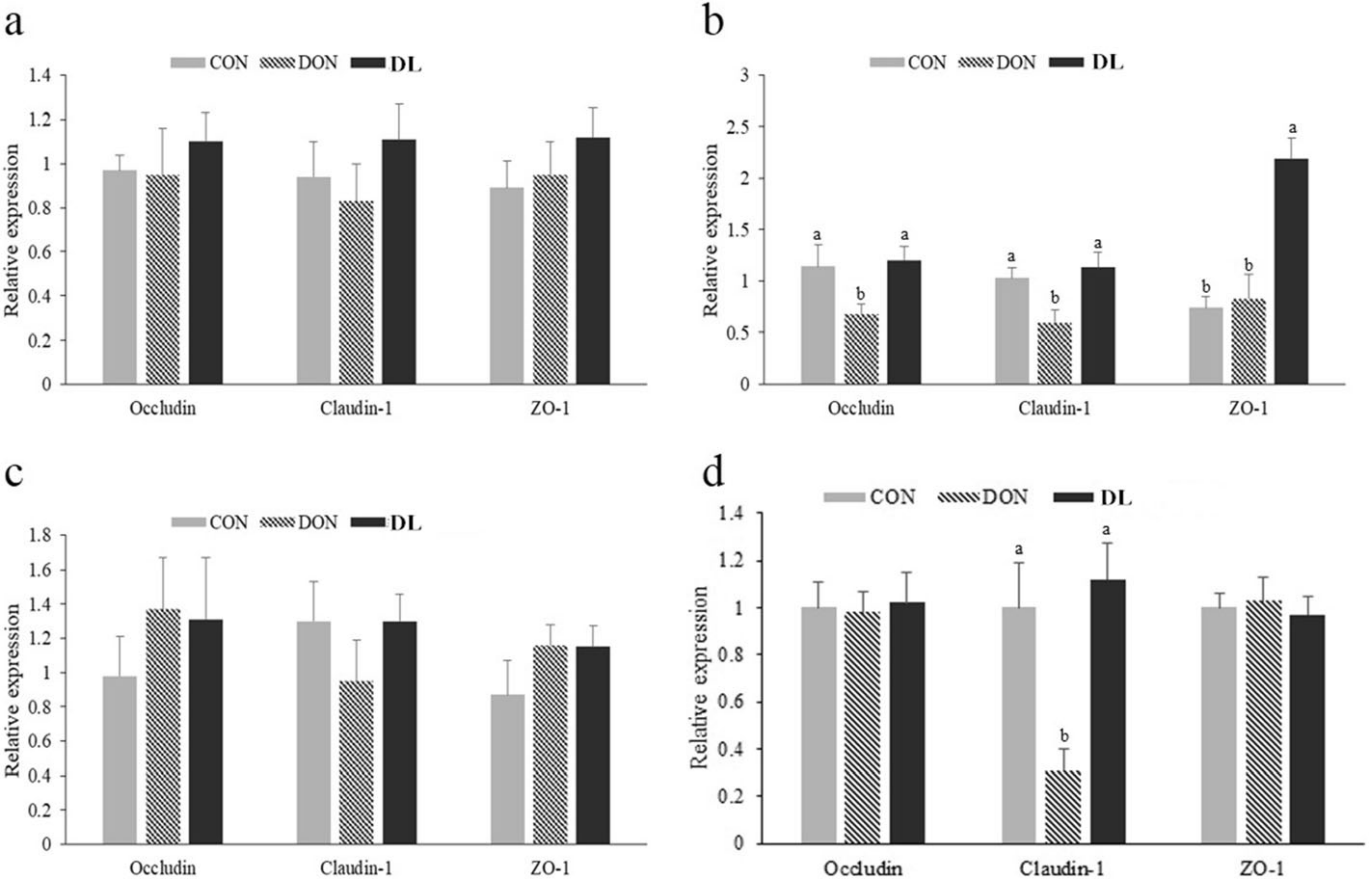
Effects of *L. plantarum* JM113 and DON on the genes expression of tight junctions in duodenum, jejunum, and ileum of 21-day-old and 42-day-old broilers. CON means the control group, DON means the deoxynivalenol group, and DL means the deoxynivalenol+ *L. plantarum* JM113 group; (**a**), (**b**), and (**c**) respectively represent the related gene expression in duodenum, jejunum, and ileum of 21-day-old broilers; and (**d**) represent the related gene expression in jejunum of 42-day-old broilers; ^a-b^ the same superscript letters upon the bar means no significant difference was detected between two groups (*P* < 0.05)

The effects of DON and *L. plantarum* JM113 on the expression of *claudin-1*, *ZO-1*, and *occludin* were investigated. In 21-day-old broilers, DON significantly reduced *claudin-1* and *occludin* mRNA expression in the jejunum (*P* = 0.044 and *P* = 0.047, Fig. 1b). However, no effects were found in *ZO-1* expression from jejunal (Fig. 1b) mRNA or the mRNA expression of the three tested genes in the duodenum and ileum (Fig. 1a and Fig. 1c). Supplementation with *L. plantarum* JM113 and DON synchronously could significantly increase jejunal *claudin-1* (*P* = 0.044), *occludin* (*P* = 0.047), and *ZO-1* (*P* < 0.001) mRNAs expression when compared with DON challenge group and have a similar expression level as the control group. Specifically, supplementing *L. plantarum* JM113 with DON significantly increased the *ZO-1* mRNA expression when compared with control group. Furthermore, in 42-day-old broilers, a similar significant change was identified in the jejunal *claudin-1* mRNAs expression (*P* < 0.05, Fig. 1d).

**Fig. 2:**
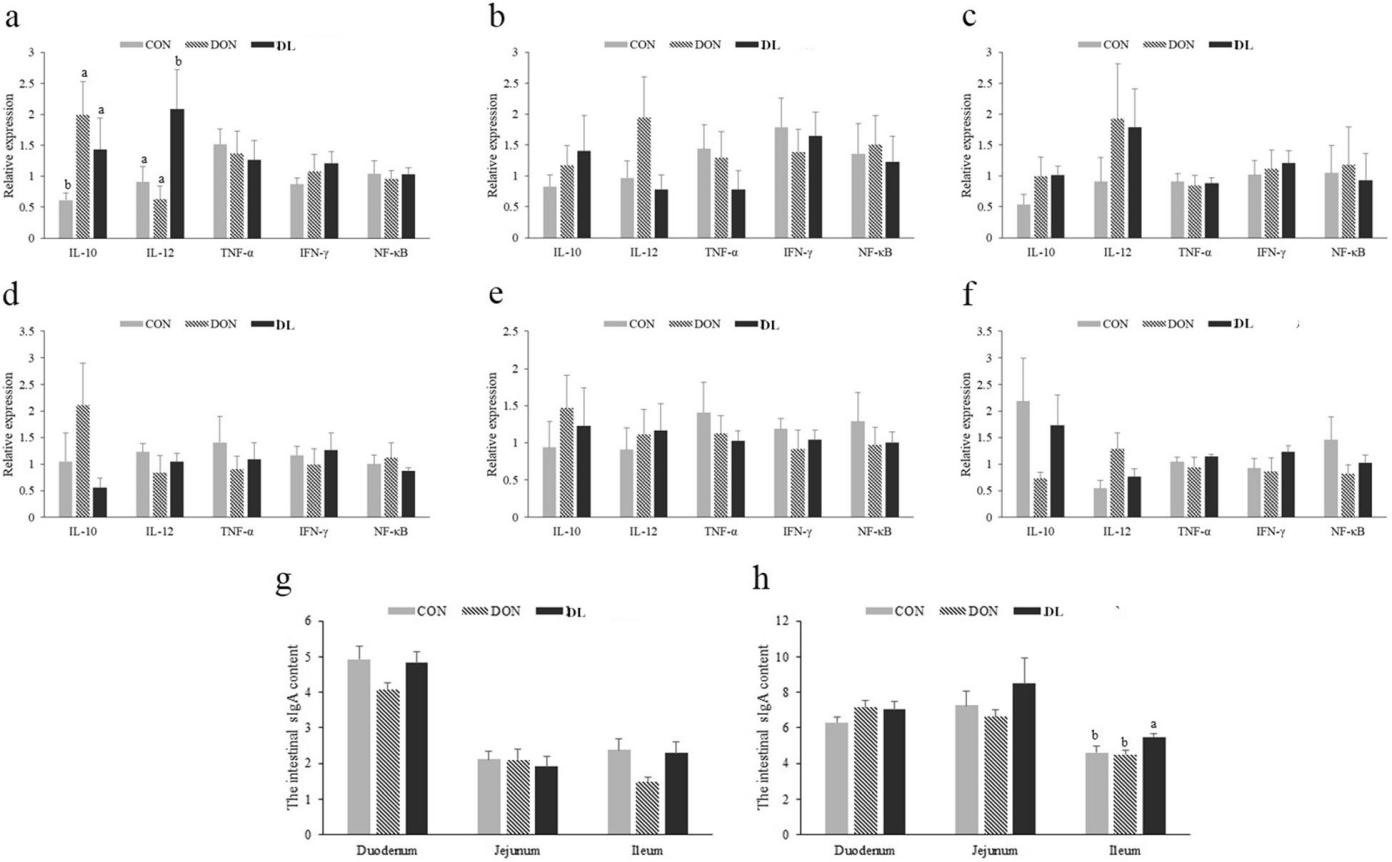
Effect of *L. plantarum* JM113 and DON on intestinal immunoreaction in 21-day-old and 42-day-old broilers. CON means control group, DON means deoxynivalenol group, and DL means the deoxynivalenol+ *L. plantarum* JM113 group; (**a)**, (**b**), and (**c**) respectively represent the gene expression of immune cytokines in duodenum, jejunum, and ileum of 21-day-old broilers; and (**d**), (**e**), and (**f**) respectively represent the gene expression of immune cytokines in duodenum, jejunum, and ileum of 42-day-old broilers; g and h respectively represent the sIgA content of 21-day-old and 42-day-old broilers; ^a-b^ the same superscript letters upon the bar means no significant difference was detected between two groups (*P* < 0.05)

The sIgA content and the expression of intestinal NF-κB and four cytokines (*IL-10*, *IL-12*, *TNF-α*, and *IFN-γ*) in the duodenum, jejunum, and ileum of 21-day-old and 42-day-old broilers was also measured. The duodenal expression of *IL-10* and *IL-12* in the 21-day-old broilers (*P* = 0.050 and *P* = 0.045) and the ileal sIgA content of 42-day-old broilers was significantly increased (*P* = 0.044) in the DL group when compared with the control group (Fig. 2a and h), which indicated that the supplementary *L. plantarum* JM113 might improve the gut immune function and further counteract the effect of DON on intestinal tissue. However, mRNA expression levels of three other mRNAs were not changed (Fig. 2). The organ indexes of 21-day-old and 42-day-old broilers were also recorded (Table 1). Compared with the control group, the development of the bursa of Fabricius was restrained in the DON group; however, supplying *L. plantarum* JM113 with DON significantly increased the relative organ index of the bursa of Fabricius (*P* = 0.016).

### *L. plantarum* JM113 repaired the impaired intestinal digestion and absorption functions induced by DON

**Fig. 3:**
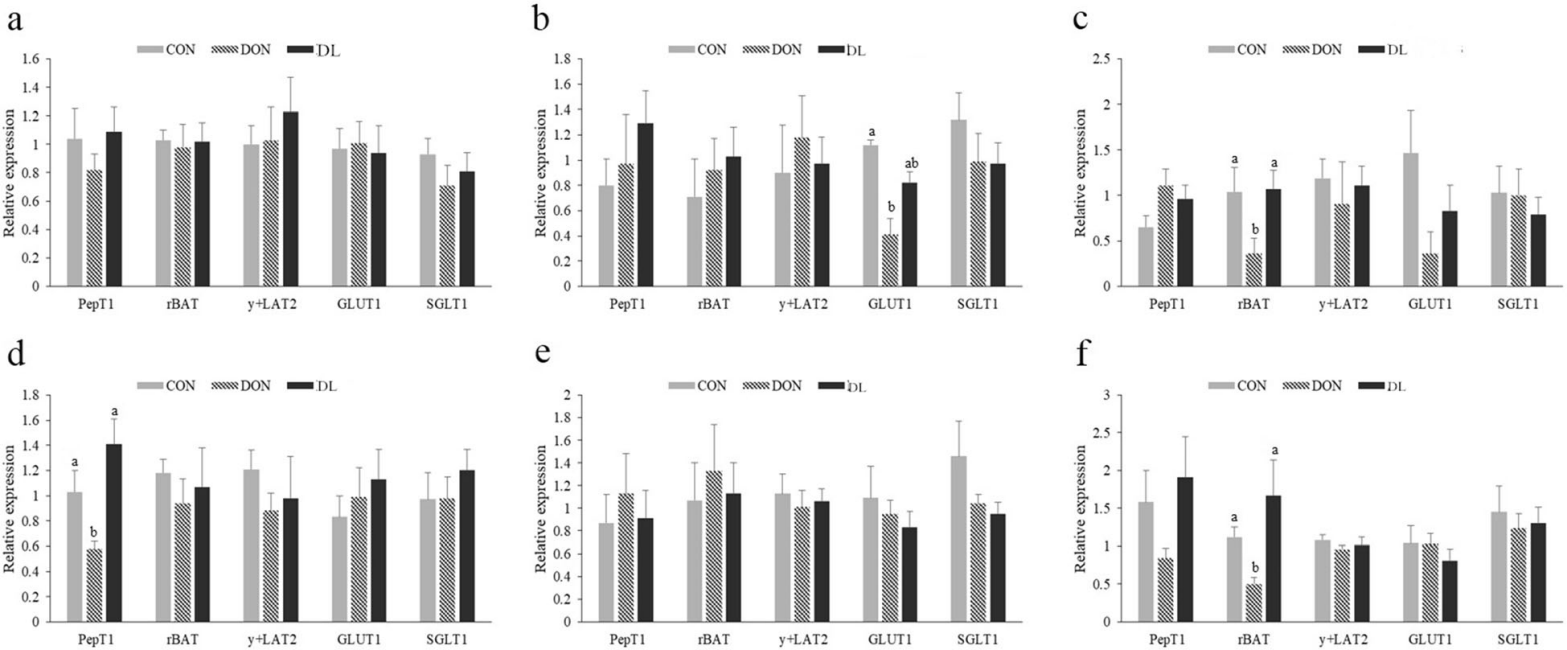
Effect of *L. plantarum* JM113 and DON on relative expression of small intestinal transporter gene of broilers. CON means control group, DON means deoxynivalenol group, and DL means the deoxynivalenol+ *L. plantarum* JM113 group; (**a**), (**b**), and (**c**) respectively represent the relative expression of small intestinal transporter gene in duodenum, jejunum, and ileum of 21-day-old broilers; and (**d**), (**e**), and (**f**) respectively represent relative expression of small intestinal transporter gene in duodenum, jejunum, and ileum of 42-day-old broilers; ^a-b^ the same superscript letters upon the bar means no significant difference was detected between two groups (*P* < 0.05)

The intestinal digestion and absorption functions of three groups were further examined. In our study, no significant changes were identified in the growth performance and the pancreatic enzymes, including amylase, chymotrypsin, and lipase (*P* > 0.05, Additional file 1: Table S3). For the intestinal absorption, the mRNA expression levels of *PepT1*, *rBAT*, *y*^+^*LAT2*, *GLUT1*, and *SGLT1* of duodenum, jejunum, and ileum in 21-day-old and 42-day-old broilers were determined. At 21 d of age, the ileal *rBAT* and jejunal *GLUT1*, which are responsible for transportation of cationic amino acids and glucose, were significantly reduced in the DON group when compared with the control and DL groups (*P* = 0.012 and *P* = 0.007, Fig. 3). At 42 d of age, compared with the control group, the mRNA expression levels of the duodenal small peptide transporter *PepT1* and the ileal amino acid transporter *rBAT* were also significantly reduced in the DON group but significantly increased in broilers supplied with both DON and *L. plantarum* JM113 (*P* = 0.013 and *P* = 0.041, Fig. 3).

### Altered transcriptomics profiles related to the administration of DON and *L. plantarum* JM113

In this study, the damaging effects of DON and the regulatory roles of *L. plantarum* JM113 mainly occurred in jejunum. To clarify the gene-network that was involved in the damaging effects of DON and the beneficial effects of *L. plantarum* JM113, further RNA sequencing was performed. Comparing between the control and DON treatments, a total of 21 up-regulated mRNAs and 28 down-regulated mRNAs were identified in the DON group (Additional file 1: Table S4). GO enrichments of the DEGs were categorized into 85 functional annotations that met the criteria of *P* < 0.05 (Additional file 1: Table S5). The results indicated that the treatment with DON induced several adverse effects including an alteration of gut integrity and the induction of an inflammatory response, including the heterotypic cell-cell adhesion, actin-mediated cell contraction, cell-cell adherens junction, and the regulation of anoikis. Specifically, the anoikis process was usually accompanied by small intestine cell shedding [41], which indicated that DON could actually have induced intestinal injury and that anoikis pathway could serve as the most important apoptotic process in the small intestinal epithelium when challenged with DON. Moreover, several biological process related to transmembrane transport and metabolism of nutrients were identified based on the DEGs, including the transport of monocarboxylic acid, organic acid, and lactate; the metabolism of glucose, ketone, thioester and nitrogen; and the biosynthesis of acetyl-CoA and fatty acid. Then, further KEGG analyses revealed that these mRNAs were significantly involved in 2 pathways, including glycosaminoglycan biosynthesis - keratan sulfate, and the nitrogen metabolism.

To illuminate the roles of *L. plantarum* JM113 in regulating gene expression, which could counteract the effect of DON on intestinal tissue, the comparison between the DL and DON treatments were further analyzed. 92 DEGs were identified (Additional file 1: Table S6), of these, 51 mRNAs were increased and 41 mRNAs were decreased in the DL group when compared with the DON group. GO and KEGG analyses were also performed and the results indicated that many of the altered genes were significantly categorized into 76 GO terms and 2 KEGG pathway (Additional file 1: Table S7). Accordingly, most of the GO terms were related to the regulation of intestinal epithelial integrity and the transmembrane transport of nutrients. Meanwhile, the significantly enriched KEGG pathways were related to phenylalanine, tyrosine and tryptophan biosynthesis, and the glycerophospholipid metabolism process.

**Fig. 4:**
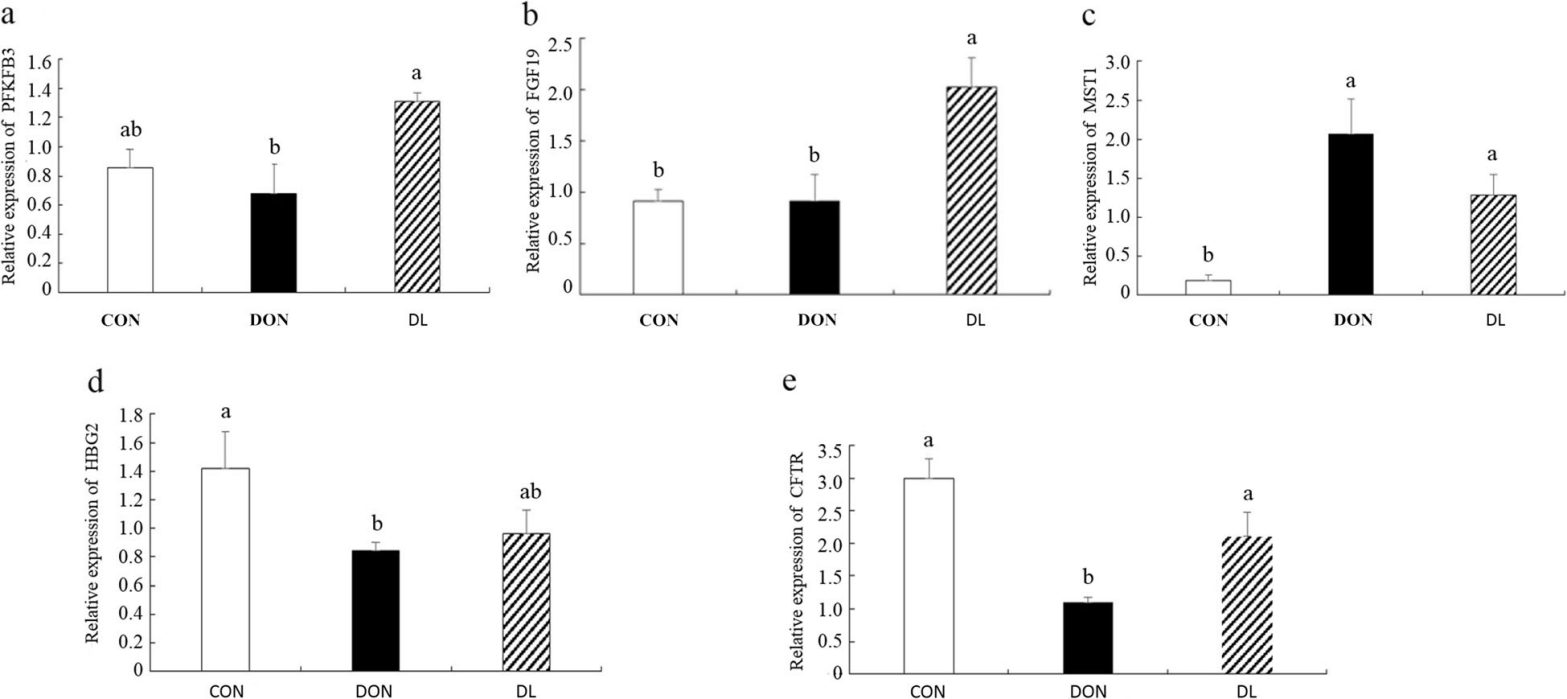
Five differentially expressed miRNAs which were validated by qRT-PCR. CON means control group, DON means deoxynivalenol group, and DL means the deoxynivalenol + *L. plantarum* JM113 group; (**a**), (**b**), (**c**), (**d**), and (**e**) respectively represent the relative gene expression of *PFKFB3*, FGF19, *MST1*,* HBG2*, and *CFTR* in jejunum of 42-day-old broilers; ^a-b^ the same superscript letters upon the bar means no significant difference was detected between two groups (*P* < 0.05)

Five of these DEGs were further verified by qRT-PCR and the results indicated that the expression of our selected mRNAs detected by qRT-PCR were consistent with the mRNA-sequencing data (Fig. 4).

### *L. plantarum* JM113 could improve the caecal microbiota when challenged with DON induced toxicity

Caecal microbiota has the most abundant microbiota in the broilers’ gut and could actually represent the whole gut microbiota of chickens [42–44]. To investigate the effect of DON on gut microbiota, the significantly altered intestinal microflora at the phylum, genus, and species levels were also analyzed statistically (Table 2). At the phylum level, the abundance of Proteobacteria was found to be significantly reduced in the DON supplemented group (*P* < 0.05). At the genus level, *Escherichia* and *Cc-115* were significantly reduced in the DON group (*P* < 0.05), and *Lactobacillus* and *Prevotella* both tended to be reduced by DON (*P* < 0.1). Moreover, at the species level, *Escherichia coli* was the only significantly altered bacteria (*P* < 0.05); meanwhile, *Ruminococcus bromii*, *Desulfovibrio C21_c20*, and *Eubacterium dolichum* all tended to be reduced by treatment with DON (*P* < 0.1).

**Table 2:** Differential cecum microbiota between control and DON groups at the phylum, genus, and species levels in 42-day-old broilers

Levels	Microbiota	CON	DON	Relative fold change [log_2_(CON/DON)]	*P*-value
Phylum	Proteobacteria	2.130%	0.949%	1.166	0.028
Genus	*Cc-115*	0.423%	0.192%	1.141	0.024
	*Escherichia*	0.432%	0.094%	2.198	0.048
	*Prevotella*	0.002%	0.000%		0.062
	*Lactobacillus*	0.076%	0.030%	1.340	0.098
Species	*Escherichia coli*	0.432%	0.094%	2.198	0.034
	*Ruminococcus bromii*	0.025%	0.005%	2.406	0.064
	*Desulfovibrio C21_c20*	0.059%	0.000%		0.076
	*Eubacterium dolichum*	0.007%	0.000%		0.088

CON means control group, DON means deoxynivalenol group

To examine the regulatory roles of *L. plantarum* JM113, the significant alterations of the caecal microbiota between the DON and DL groups were also statistically analyzed based on the phylum, genus, and species (Table 3). At the phylum level, the abundance of Bacteroidetes and Firmicutes were found to be significantly altered (*P* < 0.05), of these, Bacteroidetes was significantly increased and Firmicutes was significantly decreased in the DL group when compared with the DON group. At the genus levels, the relative abundance of *Roseburia*, *Anaerofustis*, *Anaerostipe* were significantly increased in the DL group (*P* < 0.05). The *Ruminococcus bromii* was significantly altered at the species levels in the present study (*P* < 0.05).

**Table 3:** Differential cecum microbiota between DON and DL groups at the phylum, genus, and species levels in 42-day-old broilers

Levels	Microbiota	DON	DL	Relative fold change [log_2_(DON/DL)]	*P*-value
Phylum	Bacteroidetes	9.504%	16.449%	−0.791	0.000
	Firmicutes	87.390%	80.544%	0.118	0.009
Genus	*Anaerostipes*	0.002%	0.010%	−2.459	0.000
	*Anaerofustis*	0.010%	0.045%	−2.125	0.009
	*Roseburia*	0.021%	0.127%	−2.167	0.044
Species	*Ruminococcus bromii*	0.000%	0.010%		0.008

DON means deoxynivalenol group, DL means deoxynivalenol+ *L. plantarum* JM113 group

### *L. plantarum* JM113 could improve caecal fermentation and alter caecal volatile fatty acid content

Compared with the control and DON groups, the content of propionic acid, n-Butyric acid, and total short-chain fatty acid (SCFA) were all significantly increased in the DL group (*P* < 0.05), although no significant alterations were determined between the control and DON groups (Table 4). Meanwhile, other SCFAs, including acetic acid, isobutyric acid, isopentanoic acid, and n-Pentanoic acid, were not significantly altered among the control, DON, and DL groups (Table 4).

**Table 4:** Effects of *L. plantarum* JM113 and DON on caecal volatile fatty acids contents of 42-day-old broilers

Item	Treatments^2^			SEM	*P*-value
	CON	DON	DL		
Acetic acid	117.34	108.28	175.76	13.908	0.075
Propionic acid	18.23^b^	17.45^b^	28.39^a^	1.940	0.005
Isobutyric acid	3.60	2.21	3.16	0.357	0.302
n-Butyric acid	18.97^b^	19.70^b^	34.34^a^	2.908	0.017
Isopentanoic acid	3.73	2.49	3.42	0.284	0.187
n-Pentanoic acid	5.46	4.83	6.44	0.420	0.327
Total SCFA^1^	167.32^b^	154.96^b^	251.51^a^	18.852	0.044

^1^SCFA means short-chain fatty acid

^2^CON means control group, DON means deoxynivalenol group, DL means deoxynivalenol+ *L. plantarum* JM113 group; ^a-b^ the same superscript letters upon the bar means no significant difference was detected between two groups (*P* < 0.05)

## Discussion

The damages to the intestinal histological morphology and gut integrity, and the changes in the inflammatory responses and the intestinal absorption of the amino acid, glucose, and small peptide were all identified in the present study when broilers were challenged with DON, which were widely proved by previous studies [45–49]. Strategies to alleviate the harmful effects of DON are of increasing interest and need to take into account the potential causes of the negative effects of DON contamination on poultry.

One of the many strategies to counteract the harmful effects of DON is the addition of probiotics [50, 51]. *Lactobacillus*, which improves performance, promotes the digestion and utilization, and prevents intestinal epithelial barrier dysfunction and immune responses, could be used to alleviate the harmful effects of DON [52–55]. Nevertheless, the magnitude of the effect seems to be strain dependent and host specific. *L. plantarum* JM113, which proved to have the antioxidant activities in vivo and in vitro [22], could improve the antioxidant status and protect the integrity of the intestinal barrier in broiler chickens fed diet supplemented with DON. In the present study, extra addition of *L. plantarum* JM113 can counteract the adverse effects of DON-induced damages on the intestine, as seen by the restored villus length and V: C value, and the restored intestinal tight junctions such as the mRNAs expression of *claudin-1*, *occludin*, and *ZO-1*, which were in accordance with our previous study [22]. To sum up, the results of the present study and our previous study [22] have demonstrated that addition of *L. plantarum* JM113 improved the antioxidant status and protected the integrity of the intestinal barrier in broiler chickens fed diet supplemented with DON.

Meanwhile, the restored absorption functions of the amino acid, glucose, and small peptide and the improved transmembrane transport and metabolism of phenylalanine, tyrosine, tryptophan, and glycerophospholipid, which were concluded from the qRT-PCR and the bioinformatics analysis of RNA sequencing, were also identified in the DL group. In the previous studies, the lipid metabolism and the phenylalanine, tyrosine and tryptophan metabolism were proved to be disturbed by DON contamination [5, 49, 56]. Hence, our results indicated that *L. plantarum* JM113 could improve the digestion, absorption, and metabolic functions of jejunum, especially the metabolism of lipid and amino acids, to counteract the disadvantageous effects of DON. Previous study speculated that probiotics improve the metabolic processes due to the increased microvilli height leading to an increase in the microvilli’s absorptive surface area and enabling the optimal utilization of nutrients [57]. However, this viewpoint ignored the role of probiotics in regulating the intestinal microbiota. Specifically, our previous study had proved that *L. plantarum* JM113 could effectively inhibit growth of 3 pathogens (*Escherichia coli*, *Salmonella enteritidis*, and *Salmonella typhimurium*) [22, 24], which indicated that the altered microbiota induced by *L. plantarum* JM113 could serve as the mechanism of *L. plantarum* JM113 on the protection of the intestinal digestion, absorption, and metabolic function of broiler when challenged with DON. Hence, the most important focus of the present study was on the significantly altered microbiota, which was in response to DON contamination and the protective roles of *L. plantarum* JM113.

The alterations of gut microbiota, which was induced by the feed-derived DON, were firstly studied. The abundance of Proteobacteria (phylum level), the *Escherichia, Cc-115, Lactobacillus* and *Prevotella* (genus level), as well as the *Escherichia coli*, *Ruminococcus bromii*, *Desulfovibrio C21_c20*, and *Eubacterium dolichum* (species level) were all found to be reduced by DON contamination. Among these different bacteria, the presence of Proteobacteria was favoured by an animal protein-based diet and it plays a crucial role in improving the digestibility of protein in the gut [58, 59]. The *Cc-115* belongs to Erysipelotrichaceae*,* which have been proved to take part in the digestibility of lipid and the formation of short chain fatty acids, including acetate, propionate, and butyrate [60, 61]. The abundance of *Prevotella* was positively related to the degradation of oligose, xylan, protein, and starch [62]. The abundance of *Lactobacillus* was proved to take part in the degradation of lipid and lactose and enhance the intestinal immune function [22, 63, 64]. *Ruminococcus bromii* was a keystone species for the degradation of resistant starch, which indicated that it was beneficial for the degradation of starch, reduction of chyme viscosity, and improvement in the digestibility of other nutrients [65, 66]. In conclusion, these bacteria were beneficial for improving the digestion and absorption of nutrients and were all significantly decreased by DON contamination, which indicated that DON contamination was harmful for the digestion and absorption of nutrients by influencing the abundance of these bacteria. Meanwhile, by qRT-PCR and RNA sequencing, we have proved that the digestion and absorption of amino acid, glucose, small peptide, monocarboxylic acid, organic acid, lactate, ketone, and lipid were all damaged by DON contamination, which is in accordance with the alterations in the gut microbiota. Herein, our results indicated that DON contamination could reduce the abundance of intestinal beneficial bacteria, which were beneficial for the digestion and absorption of nutrients and further influence the digestion, absorption, and metabolism of amino acids, glucose, and lipid.

As shown by previous studies, the main function of *L. plantarum* was regulating the gut microbiota and further enhancing the intestinal digestive function [52–55]. The significantly altered microbiota between the DON and DL groups were further identified. At the phylum level, Bacteroidetes was significantly increased in the DL group when compared with the DON group. The bacteria of Bacteroidetes play a fundamental role in the processing of complex molecules to simpler ones, and the increased Bacteroidetes in the gut of broilers have been proved to be positively correlated with increased growth performance [67, 68]. At the genus level, the relative abundance of *Roseburia*, *Anaerofustis*, and *Anaerostipe* were significantly increased in the DL group. *Roseburia* and *Anaerostipe* produce butyric acid by fermentation of carbohydrates, which enables a significant increase in intestinal carbohydrate absorption and energy utilization efficiency in gut [69, 70]. *Ruminococcus bromi* have been shown to assist in the degradation of resistant starches and other nutrients [65, 66], and were significantly increased in the DL group when compared with the DON group and significantly decreased in the DON group when compared with the control group. Overall, our results identified that supplementation with *L. plantarum* JM113 could increase the abundance of intestinal bacteria that improve digestion and absorption of nutrients and further enhance the digestion, absorption, and metabolism function of gut in broilers when challenged with DON contamination, which have also been observed by qRT-PCR and RNA sequencing. Moreover, determination of volatile fatty acid content has also proved that the content of propionic acid, n-Butyric acid, and total SCFA were all significantly increased in the DL group when compared with the DON group. These results indicate a better nutrient fermentability and digestibility in the DL group, which was caused by the improvement of the gut microbiota.

## Conclusion

Our results indicated that *L. plantarum* JM113 could enhance the digestion, absorption, and metabolism functions of gut when challenge with DON contamination in two main ways: by reducing the damages observed in intestinal morphology and intestinal barriers, and by increasing the abundance of beneficial bacterium and regulating the balance of gut microbiota.

## Additional file

Additional file 1: **Table S1.** Composition and nutrient level of basal diet (air-dry basis). **Table S2.** Primers used in real-time quantitative PCR. **Table S3.** Effects of *L. plantarum* JM113 and DON on activities of pancreatic digestive enzymes and growth performance of broilers. **Table S4.** Differentially expressed mRNAs of the compared group between control and DON groups in the jejunum of 42-day-old broilers. **Table S5.** Significantly enriched GO terms (*P* < 0.05) of the differentially expressed mRNAs in the DON supplement group relative to the control group. **Table S6.** Differentially expressed mRNAs of the compared group between DON and DL groups in the jejunum of 42-day-old broilers. **Table S7.** Significantly enriched GO terms and KEGG pathways (*P* < 0.05) of the differentially expressed mRNAs in the DL group relative to the DON group. (PDF 525 kb)

## Abbreviations

CFU: Colony-forming unit
DEGs: Differentially expressed genes
DON: Deoxynivalenol
ELISA: Enzyme linked immunosorbent assay
GO: Gene Ontology
HPLC: High performance liquid chromatography
KEGG: Kyoto Encyclopedia of Genes and Genomes
*L. plantarum*: *Lactobacillus plantarum*
NRC: National Research Council
OTU: Operational taxonomic unit
qRT-PCR: Quantitative real-time PCR
SCFA: Short-chain fatty acid
sIgA: Small Intestinal Secreted Immunoglobulin A
V:C: Ratio of villus height to crypt depth
VFA: Volatile fatty acid

## References

[1] Wu Q, Lohrey L, Cramer B, Yuan Z, Humpf HU (2011). Impact of physicochemical parameters on decomposition of deoxynivalenol during extrusion cooking of wheat grits. J Agric Food Chem.

[2] Tian Y, Tan Y, Liu N, Liao Y, Sun C, Wang S (2016). Functional agents to biologically control deoxynivalenol contamination in cereal grains. Front Microbiol.

[3] Awad WA, Aschenbach JR, Setyabudi F, Razzazi-Fazeli E, Böhm J, Zentek J (2007). In vitro effects of deoxynivalenol on small intestinal d-glucose uptake and absorption of deoxynivalenol across the isolated jejunal epithelium of laying hens. Poult Sci.

[4] Weaver AC, See MT, Kim SW (2014). Protective effect of two yeast based feed additives on pigs chronically exposed to deoxynivalenol and zearalenone. Toxins..

[5] Awad WA, Zentek J (2015). The feed contaminant deoxynivalenol affects the intestinal barrier permeability through inhibition of protein synthesis. Arch Toxicol.

[6] Payros D, Alassane-Kpembi I, Pierron A, Loiseau N, Pinton P, Oswald IP (2016). Toxicology of deoxynivalenol and its acetylated and modified forms. Arch Toxicol.

[7] Pinton P, Oswald IP (2014). Effect of deoxynivalenol and other type B trichothecenes on the intestine: a review. Toxins..

[8] Pestka JJ (2010). Deoxynivalenol: mechanisms of action, human exposure, and toxicological relevance. Arch Toxicol.

[9] Pestka JJ (2010). Deoxynivalenol-induced proinflammatory gene expression: mechanisms and pathological sequelae. Toxins.

[10] Awad WA, Vahjen W, Aschenbach JR, Zentek J (2011). A diet naturally contaminated with the fusarium mycotoxin deoxynivalenol down regulates gene expression of glucose transporters in the intestine of broiler chickens. Livestock Sci.

[11] Broekaert N, Devreese M, van Bergen T, Schauvliege S, De Boevre M, De Saeger S (2017). In vivo contribution of deoxynivalenol-3-β-D-glucoside to deoxynivalenol exposure in broiler chickens and pigs: oral bioavailability, hydrolysis and toxicokinetics. Arch Toxicol.

[12] Donohoe DR, Garge N, Zhang X, Sun W, O'Connell TM, Bunger MK (2011). The microbiome and butyrate regulate energy metabolism and autophagy in the mammalian colon. Cell Metab.

[13] Yang L, Liu S, Ding J, Dai R, He C, Xu K (2017). Gut microbiota co-microevolution with selection for host humoral immunity. Front Microbiol.

[14] Round JL, Mazmanian SK (2009). The gut microbiota shapes intestinal immune responses during health and disease. Nat Rev Immunol.

[15] Tremaroli V, Bäckhed F (2012). Functional interactions between the gut microbiota and host metabolism. Nature.

[16] De Filippo C, Cavalieri D, Di Paola M, Ramazzotti M, Poullet JB, Massart S, et al. Impact of diet in shaping gut microbiota revealed by a comparative study in children from Europe and rural Africa. Proc Natl Acad Sci U S A. 2010;(33):14691–6. 10.1073/pnas.1005963107 PMC2930426 20679230

[17] Jeong HG, Kang MJ, Kim HG, Oh DG, Kim JS, Lee SK (2013). Role of intestinal microflora in xenobiotic-induced toxicity. Mol Nutr Food Res.

[18] Robert H, Payros D, Pinton P, Théodorou V, Mercier-Bonin M, Oswald IP (2017). Impact of mycotoxins on the intestine: are mucus and microbiota new targets?. J Toxicol Environ Health B.

[19] Liao Y, Peng Z, Chen L, Nüssler AK, Liu L, Yang W (2018). Deoxynivalenol, gut microbiota and immunotoxicity: a potential approach?. Food Chem Toxicol.

[20] Weaver AC, See MT, Hansen JA, Kim YB, De Souza AL, Middleton TF, Kim SW (2013). The use of feed additives to reduce the effects of aflatoxin and deoxynivalenol on pig growth, organ health and immune status during chronic exposure. Toxins..

[21] Gallo A, Giuberti G, Frisvad JC, Bertuzzi T, Nielsen KF (2015). Review on mycotoxin issues in ruminants: occurrence in forages, effects of mycotoxin ingestion on health status and animal performance and practical strategies to counteract their negative effects. Toxins..

[22] Yang X, Li L, Duan Y, Yang X (2017). Antioxidant activity of *Lactobacillus plantarum* JM113 in vitro and its protective effect on broiler chickens challenged with deoxynivalenol. J Anim Sci.

[23] Brisbin JT, Gong J, Orouji S, Esufali J, Mallick AI, Parvizi P (2011). Oral treatment of chickens with lactobacilli influences elicitation of immune responses. Clin Vaccine Immunol.

[24] Feng J, Liu P, Yang X, Zhao X (2015). Screening of immunomodulatory and adhesive lactobacillus with antagonistic activities against Salmonella from fermented vegetables. World J Microb Biot.

[25] Naghi Shokri A, Ghasemi HA, Taherpour K (2017). Evaluation of Aloe vera and synbiotic as antibiotic growth promoter substitutions on performance, gut morphology, immune responses and blood constitutes of broiler chickens. Anim Sci J.

[26] Vernon SD, Shukla SK, Conradt J, Unger ER, Reeves WC (2002). Analysis of 16s rRNA gene sequences and circulating cell-free DNA from plasma of chronic fatigue syndrome and non-fatigued subjects. BMC Microbiol.

[27] Magoč T, Salzberg SL (2011). FLASH: fast length adjustment of short reads to improve genome assemblies. Bioinformatics.

[28] Caporaso JG, Kuczynski J, Stombaugh J, Bittinger K, Bushman FD, Costello EK (2010). QIIME allows analysis of high-throughput community sequencing data. Nat Methods.

[29] Edgar RC, Haas BJ, Clemente JC, Quince C, Knight R (2011). UCHIME improves sensitivity and speed of chimera detection. Bioinformatics.

[30] Haas BJ, Gevers D, Earl AM, Feldgarden M, Ward DV, Giannoukos G (2011). Chimeric 16S rRNA sequence formation and detection in sanger and 454-pyrosequenced PCR amplicons. Genome Res.

[31] Edgar RC (2013). UPARSE: highly accurate OTU sequences from microbial amplicon reads. Nat Methods.

[32] Wang Q, Garrity GM, Tiedje JM, Cole JR (2007). Naive Bayesian classifier for rapid assignment of rRNA sequences into the new bacterial taxonomy. Appl Environ Microbiol.

[33] DeSantis TZ, Hugenholtz P, Larsen N, Rojas M, Brodie EL, Keller K (2006). Greengenes, a chimera-checked 16S rRNA gene database and workbench compatible with ARB. Appl Environ Microbiol.

[34] Langmead B, Salzberg SL (2012). Fast gapped-read alignment with bowtie 2. Nat Methods.

[35] Trapnell C, Pachter L, Salzberg SL (2009). TopHat: discovering splice junctions with RNA-Seq. Bioinformatics.

[36] Trapnell C, Williams BA, Pertea G, Mortazavi A, Kwan G, Van Baren MJ (2010). Transcript assembly and quantification by RNA-Seq reveals unannotated transcripts and isoform switching during cell differentiation. Nat Biotechnol.

[37] Young MD, Wakefield MJ, Smyth GK, Oshlack A (2010). Gene ontology analysis for RNA-seq: accounting for selection bias. Genome Biol.

[38] Xie C, Mao X, Huang J, Ding Y, Wu J, Dong S (2011). KOBAS 2.0: a web server for annotation and identification of enriched pathways and diseases. Nucleic Acids Res.

[39] Livak KJ, Schmittgen TD (2001). Analysis of relative gene expression data using real-time quantitative PCR and the 2− ΔΔCT method. Methods.

[40] Shen YB, Piao XS, Kim SW, Wang L, Liu P, Yoon I (2009). Effects of yeast culture supplementation on growth performance, intestinal health, and immune response of nursery pigs. J Anim Sci.

[41] Grossmann J (2002). Molecular mechanisms of “detachment-induced apoptosis—Anoikis. Apoptosis.

[42] Polansky O, Sekelova Z, Faldynova M, Sebkova A, Sisak F, Rychlik I (2016). Important metabolic pathways and biological processes expressed by chicken cecal microbiota. Appl Environ Microbiol.

[43] Stanley D, Geier MS, Chen H, Hughes RJ, Moore RJ (2015). Comparison of fecal and cecal microbiotas reveals qualitative similarities but quantitative differences. BMC Microbiol.

[44] Torok VA, Hughes RJ, Mikkelsen LL, Perez-Maldonado R, Balding K, MacAlpine R (2011). Identification and characterization of potential performance-related gut microbiotas in broiler chickens across various feeding trials. Appl Environ Microbiol.

[45] Alassane-Kpembi I, Puel O, Pinton P, Cossalter AM, Chou TC, Oswald IP (2017). Co-exposure to low doses of the food contaminants deoxynivalenol and nivalenol has a synergistic inflammatory effect on intestinal explants. Arch Toxicol.

[46] Pasternak JA, Aiyer VIA, Hamonic G, Beaulieu AD, Columbus DA, Wilson HL (2018). Molecular and physiological effects on the small intestine of weaner pigs following feeding with Deoxynivalenol-contaminated feed. Toxins..

[47] Akbari P, Braber S, Gremmels H, Koelink PJ, Verheijden KAT, Garssen J (2014). Deoxynivalenol: a trigger for intestinal integrity breakdown. FASEB J.

[48] Maresca M, Mahfoud R, Garmy N, Fantini J (2002). The mycotoxin deoxynivalenol affects nutrient absorption in human intestinal epithelial cells. J Nutr.

[49] Constanze P, Carsten S, Pere R, Werner K, Patricia BH (2014). Organ damage and hepatic lipid accumulation in carp (cyprinus carpiol.) after feed-borne exposure to the mycotoxin, deoxynivalenol (DON). Toxins..

[50] Awad WA, Böhm J, Razzazi-Fazeli E, Ghareeb K, Zentek J (2006). Effect of addition of a probiotic microorganism to broiler diets contaminated with deoxynivalenol on performance and histological alterations of intestinal villi of broiler chickens. Poult Sci.

[51] Dänicke S, Döll S (2010). A probiotic feed additive containing spores of Bacillus subtilis and B. Licheniformis does not prevent absorption and toxic effects of the fusarium toxin deoxynivalenol in piglets. Food Chem Toxicol.

[52] Garcã­A GR, Payros D, Pinton P, Dogi CA, Laffitte J, Neves M, et al. Intestinal toxicity of deoxynivalenol is limited by *lactobacillus rhamnosus* RC007 in pig jejunum explants. Arch Toxicol 2018; 92: 983–993. 10.1007/s00204-017-2083-x 28993953

[53] Styriak I, Conkova E, Borutova R, Leng L, Mojzisova J (2007). The effect of some lactobacillus strains on deoxynivalenol biodegradation. Nutrition & Food Science.

[54] Turner PC, Wu QK, Piekkola S, Gratz S, Mykkänen H, El-Nezami H (2008). *Lactobacillus rhamnosus* strain gg restores alkaline phosphatase activity in differentiating caco-2 cells dosed with the potent mycotoxin deoxynivalenol. Food Chem Toxicol.

[55] Maidana LG, Gerez J, Pinho F, Garcia S, Apfl B (2017). Lactobacillus plantarum culture supernatants improve intestinal tissue exposed to deoxynivalenol. Exp Toxicol Pathol.

[56] Warth B, Parich A, Bueschl C, Schoefbeck D, Neumann NK, Kluger B (2015). GC-MS based targeted metabolic profiling identifies changes in the wheat metabolome following deoxynivalenol treatment. Metabolomics.

[57] Ezema C (2013). Probiotics in animal production: a review. J Vet Med Anim Health.

[58] Desai AR, Links MG, Collins SA, Mansfield GS, Drew MD, Kessel AGV (2012). Effects of plant-based diets on the distal gut microbiome of rainbow trout (oncorhynchus mykiss). Aquaculture.

[59] Apper E, Weissman D, Respondek F, Guyonvarch A, Baron F, Boisot P (2016). Hydrolysed wheat gluten as part of a diet based on animal and plant proteins supports good growth performance of asian seabass (lates calcarifer), without impairing intestinal morphology or microbiota. Aquaculture.

[60] Kaakoush NO (2015). Insights into the role of erysipelotrichaceae in the human host. Frontiers in Cellular & Infection Microbiology.

[61] Lecomte V, Kaakoush NO, Maloney CA, Raipuria M, Huinao KD, Mitchell HM (2015). Changes in gut microbiota in rats fed a high fat diet correlate with obesity-associated metabolic parameters. PLoS One.

[62] Dodd D, Moon YH, Swaminathan K, Mackie RI, Cann IK (2010). Transcriptomic analyses of xylan degradation by prevotella bryantii and insights into energy acquisition by xylanolytic bacteroidetes. J Biol Chem.

[63] Chen J, Deng YX, Zhang B, Liu DY, Hui XU (2017). Effect of cla producing lactobacillus on gut immune function in mice. Food Science & Technology.

[64] Wang H, Ni X, Qing X, Zeng D, Luo M, Liu L, et al. Live probiotic lactobacillus johnsonii bs15 promotes growth performance and lowers fat deposition by improving lipid metabolism, intestinal development, and gut microflora in broilers. Front Microbiol. 2017;8 10.3389/fmicb.2017.01073. 10.3389/fmicb.2017.01073 PMC5466961 28659893

[65] Abell GCJ, Cooke CM, Bennett CN, Conlon MA, Mcorist AL (2010). Phylotypes related to ruminococcus bromii are abundant in the large bowel of humans and increase in response to a diet high in resistant starch. FEMS Microbiol Ecol.

[66] Ze X, Duncan SH, Louis P, Flint HJ (2013). Ruminococcus bromii is a keystone species for the degradation of resistant starch in the human colon. ISME J.

[67] Thomas F, Hehemann JH, Rebuffet E, Czjzek M, Michel G (2011). Environmental and gut bacteroidetes: the food connection. Front Microbiol.

[68] Stanley D, Geier MS, Denman SE, Haring VR, Crowley TM, Hughes RJ (2013). Identification of chicken intestinal microbiota correlated with the efficiency of energy extraction from feed. Vet Microbiol.

[69] Louis P, Young P, Holtrop G, Flint HJ (2010). Diversity of human colonic butyrate-producing bacteria revealed by analysis of the butyryl-coa:acetate coa-transferase gene. Environ Microbiol.

[70] Sh PSD, Hold GL, Stewart CS, Flint HJ (2002). The microbiology of butyrate formation in the human colon. FEMS Microbiol Lett.

