# Supplementary material for: Intestinal toxicity of deoxynivalenol is limited by supplementation with Lactobacillus plantarum JM113 and consequentially altered gut microbiota in broiler chickens
Source: J Anim Sci Biotechnol. 2018 Oct 8;9:74. doi: 10.1186/s40104-018-0286-5 (PMC6174567; doi:10.1186/s40104-018-0286-5)
Supplement: Supplementary file 1 — Table S1. Composition and nutrient level of basal diet (air-dry basis). Table S2. Primers used in real-time quantitative PCR. Table S3. Effects of L. plantarum JM113 and DON on activities of pancreatic digestive enzymes and growth performance of broilers. Table S4. Differentially expressed mRNAs of the compared group between control and DON groups in the jejunum of 42-day-old broilers. Table S5. Significantly enriched GO terms (P < 0.05) of the differentially expressed mRNAs in the DON supplement group relative to the control group. Table S6. Differentially expressed mRNAs of the compared group between DON and DL groups in the jejunum of 42-day-old broilers. Table S7. Significantly enriched GO terms and KEGG pathways (P < 0.05) of the differentially expressed mRNAs in the DL group relative to the DON group. (PDF 525 kb) [file 40104_2018_286_MOESM1_ESM.pdf]

Supporting information

Table S1 Composition and nutrient level of basal diet (air-dry basis).

Table S2 Primers used in real-time quantitative PCR.

Table S3 Effects of *L. plantarum* JM113 and DON on activities of pancreatic digestive enzymes and growth performance of broilers.

Table S4. Differentially expressed mRNAs of the compared group between control and DON groups in the jejunum of 42-day-old broilers.

Table S5 Significantly enriched GO terms (*P* < 0.05) of the differentially expressed mRNAs in the DON supplement group relative to the control group.

Table S6. Differentially expressed mRNAs of the compared group between DON and DL groups in the jejunum of 42-day-old broilers.

Table S7 Significantly enriched GO terms and KEGG pathways (*P* < 0.05) of the differentially expressed mRNAs in the DL group relative to the DON group.

Table S1 Composition and nutrient level of basal diet (air-dry basis)

Ingredients, %			Nutrient Levels[2]		
Items	1 to 3 weeks	4 to 6 weeks	Items	1 to 3 weeks	4 to 6 weeks
Corn	56.00	53.50	ME, MJ/kg	12.14	12.77
Soybean meal	26.60	22.00	Crude protein, %	21.25	19.30
Four	6.00	8.00	Calcium, %	0.90	1.16
Corn gluten meal	4.20	1.70	TP, %	0.65	0.62
DDGS-28	3.00	5.00	AP, %	0.43	0.35
$CaHPO_4$	1.65	0.28	EE, %	2.61	6.39
$CaCO_3$	1.08	1.01	NaCl, %	0.26	0.29
L-Lysine H_2SO_4	0.33	0.24	Lys, %	1.24	1.08
Premix[1]	0.45	0.45	Met, %	0.52	0.50
Salt	0.20	0.20	Met+Cys, %	0.86	0.79
DL-Methionine	0.19	0.21	Thr, %	0.87	0.70
Thr	0.10	0.11			
$NaHCO_3$	0.10	0.10			
Choline chloride	0.08	0.08			
Phytase	0.02	0.02			
Mixed oil	-	2.60			
Meat and bone meal	-	2.00			
Instant noodle	-	2.00			
Feather meal	-	0.50			
Total	100.00	100.00			

Note： [1] The premix provided the following nutrients per kg of diets： VA 11600 IU、VD_3 3360 IU、VK_3 4.02 mg、VB_1 2.2 mg、VB_2 7.3 mg、VB_6 4.9 mg、VB_{12} 0.014 mg、Cu 8.4 mg、Zn 49.5 mg、Fe 54.0 mg、Mn 150.0 mg；

[2]The contents of CP, Ca, TP and EE were measured by feed proximity analysis method and others were calculated.

Table S2 Primers used in real-time quantitative PCR

Gene name/abbrevietion	Primer sequences (5’to 3’)
β-actin	F:ATTGTCCACCGCAAATGCTTC<br>R:AAATAAAGCCATGCCAATCTCGTC
solute carrier family 15 member 1 (*SLC15A1*; also known as *PepT1*)	F: TTCCCATGGAGTCAACAGGC<br>R: CTAGAAACAATGCCGGCTGC
solute carrier family 3 member 1 (*SLC3A1*; *rBAT*)	F: ACACCAGTGACAAGCACCAA<br>R: ACACTCACCCAGTTGTTGGG
Sodium-glucose cotransporter 1 (*SGLT1*)	F:AGCATTTCAGCATGGTGTGTCTTC<br>R:GATGCTCCTATCTCAGGGCAGTTC
solute carrier family 2 member 1(*SLC2A1*; also known as Glucose transporter 1, *GLUT1*)	F: GCAAGATGACAGCTCGCCT<br>R: GTCTTCAATCACCTTCTGCGG
y^+L amino acid transporter 2 (*y^+LAT2*)	F:CCTGATAGTAGGCAACAT<br>R:AGAACAAGGCAGAGTAGAG
Claudin-1(*CLDN1*)	F: CATCATTGCAGGTCTGTCAGC<br>R: AAAGGGTCATAGAAGGCCCG
occludin (*OCLD*)	F:TTAACCCCTCTTGTGCCGTT<br>R:ATTGAAGTCCTTCTTTCCTGCC
tight junction protein 1(*TJP1*, also known as zona occludens 1, ZO1)	F:GACTGCGCCAAGTACAGATGC<br>R:CACTCGGTGGCTCCGTAGAAC
TNF-α	F: TGTGTATGTGCAGCAACCCGTAGT<br>R: GGCATTGCAATTTGGACAGAAGT
IFN-γ	F: ACTGAGCCAGATTGTTTCGATGT<br>R: TGCCATTAGCAATTGCATCTCCT
IL-10	F: CGGGAGCTGAGGGTGAA<br>R: GTGAAGAAGCGGTGACAGC
IL-12	F: AGACTCCAATGGGCAAATGA<br>R: CTCTTCGGCAAATGGACAGT
MST1	F: TACACCATGGATCCACGCAC<br>R: CGAATGTCACTGCATCTGCG
CFTR	F: TGATGCTGAAAGCATGGGTACT<br>R: GCATTTATTTGTGCCGCTTTTTGA
Fgfl19	F: CCGCAGAGTCTGTTGGAGAT<br>R: GTTGTAGCCGTCTGGACGAA
PFKFB3	F: CGTTCCGCAAAGGTGGC<br>R: ATCACAGTGGGGGAATTGGC

HBG2	F: CGTCCTTTGGGAACCTCTCC<br>R: CCAGGAGCCTGAAGTTCTCG

Table S3 Effects of L. *plantarum* JM113 and DON on activities of pancreatic digestive enzymes and growth performance of broilers.

Item		Treatment[1]			SEM	*P* value
		CON	DON	DL		
1 to 21d	pancreatic amylase, U/mgprot	4936.99	4556.25	3878.48	644.27	0.839
	chymotrypsin, U/mgprot	560.11	942.69	469.78	93.66	0.072
	pancreatic lipase, U/mgprot	3.65	4.56	3.04	0.82	0.727
	Average body weight gain, g/d	28.29	28.84	29.16	0.42	0.719
	Average daily feed intake, g/d	43.20	44.30	43.92	0.71	0.827
	Feed intake: Body weight gain	1.52	1.53	1.50	0.01	0.791
22 to 42d	pancreatic amylase, U/mgprot	2135.22	2685.35	2376.05	234.84	0.648
	chymotrypsin, U/mgprot	408.05	495.46	575.51	48.35	0.408
	pancreatic lipase, U/mgprot	0.45	2.15	0.69	0.31	0.064
	Average body weight gain, g/d	61.06	59.62	57.97	1.23	0.623
	Average daily feed intake, g/d	126.48	128.63	126.11	1.13	0.649
	Feed intake: Body weight gain	2.08	2.17	2.18	0.04	0.520

[1] CON means the control group, DON means the deoxynivalenol group, DL means the deoxynivalenol+ *L. plantarum* JM113 group.

Table S4. Differentially expressed mRNAs of the compared group between the control and DON groups in the jejunum of 42-day-old broilers.

Gene ID	Gene Name	DON	CON	Relative fold change [$\log_2$(DON/CON)]	*P* value
ENSGALG00000000162	*DMB1*	3499.74	12052.60	-1.78	0.038
ENSGALG00000002390		143.53	441.76	-1.62	0.000
ENSGALG00000002453	*ABCA7*	132.26	63.06	1.07	0.002
ENSGALG00000002845	*CTNNA3*	8.45	23.21	-1.46	0.048
ENSGALG00000004074	*KCNS1*	6.13	27.80	-2.18	0.003
ENSGALG00000005672	*SLC6A7*	16.54	37.90	-1.20	0.036
ENSGALG00000006324		1335.32	2682.30	-1.01	0.000
ENSGALG00000006654		77.63	35.80	1.12	0.046
ENSGALG00000007596		45.78	322.41	-2.82	0.042
ENSGALG00000007701		1258.88	2879.23	-1.19	0.020
ENSGALG00000008323		1006.68	2089.80	-1.05	0.006
ENSGALG00000009457	*PFN3*	48.78	17.17	1.51	0.004
ENSGALG00000009497	*AVPR2*	2.26	13.45	-2.57	0.045
ENSGALG00000009700	*PDK4*	5835.45	2790.48	1.06	0.000
ENSGALG00000010583	*VIT*	11.01	56.49	-2.36	0.001
ENSGALG00000012531		4405.43	1917.33	1.20	0.012
ENSGALG00000013304	*SLC5A12*	95.35	2517.27	-4.72	0.029
ENSGALG00000013503	*FAM149A*	79.35	165.32	-1.06	0.018
ENSGALG00000014108		13.05	42.15	-1.69	0.018
ENSGALG00000014883	*MCM9*	149.93	71.70	1.06	0.026
ENSGALG00000015774	*CYYR1*	131.50	61.86	1.09	0.043
ENSGALG00000016837	*MYO16*	139.15	47.54	1.55	0.007
ENSGALG00000021357		26.95	59.92	-1.15	0.019
ENSGALG00000022202		43.18	12.75	1.76	0.019
ENSGALG00000024007		32.31	65.33	-1.02	0.011
ENSGALG00000024365		2.19	17.54	-3.00	0.019
ENSGALG00000024372		69192.25	155830.68	-1.17	0.035
ENSGALG00000025960		5.66	19.10	-1.75	0.043
ENSGALG00000026460		94.20	37.20	1.34	0.036
ENSGALG00000026466		946.46	457.74	1.05	0.005
ENSGALG00000026603		2.08	15.48	-2.90	0.006
ENSGALG00000026738		64.37	30.19	1.09	0.010
ENSGALG00000026798		342.74	144.92	1.24	0.044
ENSGALG00000026812		10.00	24.44	-1.29	0.033
ENSGALG00000027045		63.03	22.49	1.49	0.012

ENSGALG00000027164		464.83	209.48	1.15	0.045
ENSGALG00000027253		91.25	39.66	1.20	0.003
ENSGALG00000027425		349.98	153.39	1.19	0.000
ENSGALG00000027546	*BZFP1*	84.80	217.27	-1.36	0.018
ENSGALG00000027624		78.64	325.56	-2.05	0.008
ENSGALG00000027694		4562.78	2122.64	1.10	0.000
ENSGALG00000027944		480.58	233.26	1.04	0.010
ENSGALG00000028245		3.96	52.47	-3.73	0.000
ENSGALG00000028339		61.65	149.18	-1.27	0.031
ENSGALG00000028399		0.47	1020.48	-11.07	0.000
ENSGALG00000028500		18.16	41.35	-1.19	0.031
ENSGALG00000029079		383.69	154.96	1.31	0.000
ENSGALG00000024420		0.00	6.64	NA	0.015
ENSGALG00000021862		7.78	0.00	NA	0.017

Note: CON means control group, DON means deoxynivalenol group.

Table S5 Significantly enriched GO terms (*P* < 0.05) of the differentially expressed mRNAs in the DON supplement group relative to the control group.

ID	Term	Input number	Background number	*P* Value
GO:0008028	monocarboxylic acid transmembrane transporter activity	2	8	0.000
GO:0006342	chromatin silencing	2	22	0.002
GO:0045814	negative regulation of gene expression, epigenetic	2	23	0.002
GO:0000786	nucleosome	2	25	0.002
GO:0044815	DNA packaging complex	2	27	0.002
GO:0015718	monocarboxylic acid transport	2	29	0.003
GO:0005342	organic acid transmembrane transporter activity	2	32	0.003
GO:0046943	carboxylic acid transmembrane transporter activity	2	32	0.003
GO:0015293	symporter activity	2	37	0.004
GO:0016458	gene silencing	2	38	0.004
GO:0040029	regulation of gene expression, epigenetic	2	40	0.005
GO:0032993	protein-DNA complex	2	45	0.006
GO:0015291	secondary active transmembrane transporter activity	2	60	0.010
GO:0046942	carboxylic acid transport	2	62	0.011
GO:0015711	organic anion transport	2	66	0.012
GO:0086091	regulation of heart rate by cardiac conduction	1	5	0.015
GO:1903115	regulation of actin filament-based movement	1	5	0.015
GO:0015721	bile acid and bile salt transport	1	5	0.015
GO:0086005	ventricular cardiac muscle cell action potential	1	5	0.015
GO:0098900	regulation of action potential	1	5	0.015
GO:0000790	nuclear chromatin	2	78	0.016

GO:0015129	lactate transmembrane transporter activity	1	6	0.017
GO:0042304	regulation of fatty acid biosynthetic process	1	6	0.017
GO:0006086	acetyl-CoA biosynthetic process from pyruvate	1	6	0.017
GO:0015727	lactate transport	1	6	0.017
GO:0086002	cardiac muscle cell action potential involved in contraction	1	6	0.017
GO:0002474	antigen processing and presentation of peptide antigen via MHC class I	1	6	0.017
GO:0006085	acetyl-CoA biosynthetic process	1	6	0.017
GO:0035873	lactate transmembrane transport	1	6	0.017
GO:0042555	MCM complex	1	6	0.017
GO:0042611	MHC protein complex	1	6	0.017
GO:0086003	cardiac muscle cell contraction	1	7	0.019
GO:0034113	heterotypic cell-cell adhesion	1	7	0.019
GO:0061337	cardiac conduction	1	7	0.019
GO:0051193	regulation of cofactor metabolic process	1	7	0.019
GO:0086001	cardiac muscle cell action potential	1	7	0.019
GO:1901618	organic hydroxy compound transmembrane transporter activity	1	7	0.019
GO:0051196	regulation of coenzyme metabolic process	1	7	0.019
GO:0032970	regulation of actin filament-based process	2	86	0.019
GO:0022804	active transmembrane transporter activity	2	91	0.022
GO:0045124	regulation of bone resorption	1	8	0.022
GO:0006084	acetyl-CoA metabolic process	1	8	0.022
GO:0035384	thioester biosynthetic process	1	8	0.022
GO:0071616	acyl-CoA biosynthetic process	1	8	0.022
GO:0003779	actin binding	2	94	0.023
GO:0055117	regulation of cardiac muscle contraction	1	9	0.024

GO:0003823	antigen binding	1	9	0.024
GO:0046850	regulation of bone remodeling	1	9	0.024
GO:0006942	regulation of striated muscle contraction	1	9	0.024
GO:0045296	cadherin binding	1	9	0.024
GO:0006820	anion transport	2	100	0.026
GO:0005343	organic acid:sodium symporter activity	1	10	0.027
GO:0043276	anoikis	1	11	0.029
GO:0014704	intercalated disc	1	11	0.029
GO:0048002	antigen processing and presentation of peptide antigen	1	11	0.029
GO:2000209	regulation of anoikis	1	11	0.029
GO:2000811	negative regulation of anoikis	1	11	0.029
GO:0034103	regulation of tissue remodeling	1	11	0.029
GO:0019217	regulation of fatty acid metabolic process	1	11	0.029
GO:0070252	actin-mediated cell contraction	1	12	0.031
GO:0000785	chromatin	2	112	0.031
GO:0005913	cell-cell adherens junction	1	13	0.034
GO:0045453	bone resorption	1	13	0.034
GO:0046982	protein heterodimerization activity	2	118	0.035
GO:0044454	nuclear chromosome part	2	119	0.035
GO:0032233	positive regulation of actin filament bundle assembly	1	14	0.036
GO:0044291	cell-cell contact zone	1	14	0.036
GO:0000228	nuclear chromosome	2	123	0.037
GO:0002027	regulation of heart rate	1	16	0.041
GO:0030048	actin filament-based movement	1	16	0.041
GO:0035637	multicellular organismal signaling	1	16	0.041
GO:0010906	regulation of glucose metabolic process	1	16	0.041

GO:0010565	regulation of cellular ketone metabolic process	1	16	0.041
GO:0006637	acyl-CoA metabolic process	1	16	0.041
GO:0035383	thioester metabolic process	1	16	0.041
GO:0034109	homotypic cell-cell adhesion	1	17	0.043
GO:0015370	solute:sodium symporter activity	1	18	0.046
GO:0019395	fatty acid oxidation	1	18	0.046
GO:0007044	cell-substrate junction assembly	1	18	0.046
GO:0010675	regulation of cellular carbohydrate metabolic process	1	19	0.048
GO:0046849	bone remodeling	1	19	0.048
GO:0006885	regulation of pH	1	19	0.048
GO:0060048	cardiac muscle contraction	1	19	0.048
GO:0034440	lipid oxidation	1	19	0.048
GO:0008286	insulin receptor signaling pathway	1	19	0.048

Table S6. Differentially expressed mRNAs of the compared group between DON and DL groups in the jejunum of 42-day-old broilers.

Gene ID	Gene Name	DL	DON	Relative fold change [log2(DL/DON)]	*P* value
ENSGALG00000001063		182.16	438.63	-1.27	0.024
ENSGALG00000001505		587.70	273.39	1.10	0.000
ENSGALG00000001766		27.06	10.81	1.32	0.033
ENSGALG00000002319	*TEKT2*	36.51	16.45	1.15	0.042
ENSGALG00000002480		41.40	18.47	1.16	0.016
ENSGALG00000002513		1412.26	568.06	1.31	0.000
ENSGALG00000002595	*GPX4*	1601.88	4808.59	-1.59	0.050
ENSGALG00000002722	*MST1*	761.57	2006.62	-1.40	0.000
ENSGALG00000002893	*STC2*	462.10	178.35	1.37	0.025
ENSGALG00000002931	*REG4*	2864.79	933.44	1.62	0.000
ENSGALG00000003594	*ANK1*	35.26	14.16	1.32	0.011
ENSGALG00000003859	*KRT222*	57.64	27.02	1.09	0.009
ENSGALG00000004050		21.53	8.50	1.34	0.049
ENSGALG00000004660	*TRPV3*	61.91	27.84	1.15	0.027
ENSGALG00000005149		52.44	16.37	1.68	0.003
ENSGALG00000005178		1598.92	658.06	1.28	0.000
ENSGALG00000005349		38.53	17.46	1.14	0.033
ENSGALG00000005933	*SLC5A11*	2767.68	1134.75	1.29	0.001
ENSGALG00000005941		68.88	150.73	-1.13	0.045
ENSGALG00000005957	*SLC6A14*	167.42	343.82	-1.04	0.046
ENSGALG00000006490	*SCN3B*	49.19	18.64	1.40	0.042
ENSGALG00000006662	*BPIFB3*	21.59	9.04	1.26	0.043
ENSGALG00000006720		29.45	109.63	-1.90	0.011
ENSGALG00000007311	*CLDN2*	1883.81	3981.11	-1.08	0.004
ENSGALG00000007419	*MASP1*	129.40	274.47	-1.08	0.001
ENSGALG00000007526		11949.46	31321.29	-1.39	0.019
ENSGALG00000008135	*SATB2*	391.14	94.32	2.05	0.004
ENSGALG00000008323		1783.48	849.79	1.07	0.000
ENSGALG00000008598		537.86	60.03	3.16	0.013
ENSGALG00000008874	*SLC13A1*	1448.13	690.81	1.07	0.004
ENSGALG00000008992		3246.05	1614.55	1.01	0.027
ENSGALG00000010032		24937.39	7394.76	1.75	0.000
ENSGALG00000010748	*EXO1*	40.79	88.99	-1.13	0.002
ENSGALG00000010909	*PRKG2*	537.26	170.98	1.65	0.028
ENSGALG00000011129		42.43	13.10	1.70	0.031
ENSGALG00000011633	*SLC5A8*	221.78	1012.23	-2.19	0.000
ENSGALG00000011657	*EAF2*	124.03	414.07	-1.74	0.005
ENSGALG00000011940		947.28	401.98	1.24	0.001
ENSGALG00000012298	*SLC39A8*	40.55	19.74	1.04	0.031
ENSGALG00000012754	*PAH*	41.09	6.49	2.66	0.032

ENSGALG00000012834	*AKR1D1*	473.66	1347.98	-1.51	0.014
ENSGALG00000013101		4596.62	1964.59	1.23	0.029
ENSGALG00000013304	*SLC5A12*	996.93	80.48	3.63	0.001
ENSGALG00000013422	*RHNO1*	11.97	34.06	-1.51	0.009
ENSGALG00000013503	*FAM149A*	154.11	67.02	1.20	0.003
ENSGALG00000013720	*FBXO15*	20.97	50.95	-1.28	0.004
ENSGALG00000014242		30.98	64.84	-1.07	0.009
ENSGALG00000014466	*PIANP*	23.80	10.38	1.20	0.042
ENSGALG00000015347	*ZPLD1*	28.61	127.42	-2.16	0.001
ENSGALG00000015389	*TMPRSS7*	1067.74	491.76	1.12	0.000
ENSGALG00000015495	*POU1F1*	15.94	0.97	4.03	0.000
ENSGALG00000015722	*TMED11*	31.69	13.32	1.25	0.017
ENSGALG00000015774	*CYYR1*	28.34	111.06	-1.97	0.001
ENSGALG00000015985		26.60	55.36	-1.06	0.017
ENSGALG00000016761	*LYG2*	330.68	1806.08	-2.45	0.000
ENSGALG00000018981		14.75	3.72	1.99	0.032
ENSGALG00000019060	*MMP27*	10.44	26.92	-1.37	0.042
ENSGALG00000019211	*MAEL*	582.91	286.96	1.02	0.010
ENSGALG00000019221		41.74	97.33	-1.22	0.001
ENSGALG00000019837		384.33	910.65	-1.24	0.001
ENSGALG00000020796		68.31	184.17	-1.43	0.031
ENSGALG00000022250		22.31	55.24	-1.31	0.005
ENSGALG00000022751		24.54	52.79	-1.11	0.034
ENSGALG00000023035		5.95	35.56	-2.58	0.017
ENSGALG00000023424		3791.45	10800.77	-1.51	0.008
ENSGALG00000023761		295.06	118.84	1.31	0.021
ENSGALG00000024484		3.49	14.47	-2.05	0.032
ENSGALG00000025905		206.30	421.01	-1.03	0.038
ENSGALG00000026020		50.41	25.15	1.00	0.015
ENSGALG00000026145		232.02	17.23	3.75	0.000
ENSGALG00000026153	*FOXO6*	24.27	8.32	1.54	0.015
ENSGALG00000026262		2290.33	966.81	1.24	0.000
ENSGALG00000026460		26.56	79.50	-1.58	0.027
ENSGALG00000026603		11.85	1.76	2.75	0.046
ENSGALG00000026798		63.55	289.36	-2.19	0.001
ENSGALG00000027790		8.69	0.48	4.18	0.011
ENSGALG00000027868	*AMER2*	109.25	45.55	1.26	0.044
ENSGALG00000027901		0.77	11.89	-3.95	0.032
ENSGALG00000027956		5.41	101.56	-4.23	0.007
ENSGALG00000027974		15.32	4.92	1.64	0.033
ENSGALG00000028284	*PTX3*	25.45	8.03	1.66	0.012
ENSGALG00000028339		110.41	52.04	1.09	0.006
ENSGALG00000028376	*FGF19*	806.83	281.71	1.52	0.000
ENSGALG00000028416		16.44	35.63	-1.12	0.034

ENSGALG00000028500		39.43	15.34	1.36	0.009
ENSGALG00000028592		64.23	29.20	1.14	0.004
ENSGALG00000028669		9.28	23.02	-1.31	0.046
ENSGALG00000028990		1899.00	5002.80	-1.40	0.023
ENSGALG00000029168	*CLTRN*	39.14	78.44	-1.00	0.035
ENSGALG00000009117		0.00	236.60	NA	0.044
ENSGALG00000010486		0.00	5.15	NA	0.037
ENSGALG00000028505		0.00	77.63	NA	0.000

Note: DON means deoxynivalenol group, DL means deoxynivalenol+ *L. plantarum* JM113 group.

Table S7 Significantly enriched GO terms (*P* < 0.05) of the differentially expressed mRNAs in the DL group relative to the DON group.

ID	GO Term	Input number	Background number	*P* Value
GO:0015293	symporter activity	4	37	0.000
GO:0015291	secondary active transmembrane transporter activity	4	60	0.000
GO:0022804	active transmembrane transporter activity	4	91	0.001
GO:0005576	extracellular region	12	1028	0.005
GO:0015294	solute:cation symporter activity	2	29	0.011
GO:0015081	sodium ion transmembrane transporter activity	2	31	0.012
GO:0005342	organic acid transmembrane transporter activity	2	32	0.013
GO:0046943	carboxylic acid transmembrane transporter activity	2	32	0.013
GO:0030030	cell projection organization	5	281	0.014
GO:0006820	anion transport	3	100	0.015
GO:0048858	cell projection morphogenesis	4	197	0.019
GO:0007409	axonogenesis	3	111	0.020
GO:0032990	cell part morphogenesis	4	203	0.020
GO:0098656	anion transmembrane transport	2	43	0.022
GO:0061564	axon development	3	118	0.023
GO:0048667	cell morphogenesis involved in neuron differentiation	3	124	0.026
GO:0040011	locomotion	5	329	0.026
GO:0008509	anion transmembrane transporter activity	2	50	0.028
GO:0043623	cellular protein complex assembly	3	130	0.029
GO:1902358	sulfate transmembrane transport	1	5	0.030
GO:0051954	positive regulation of amine transport	1	5	0.030
GO:0010259	multicellular organism aging	1	5	0.030
GO:0008540	proteasome regulatory particle, base subcomplex	1	5	0.030

GO:0046326	positive regulation of glucose import	1	5	0.030
GO:0010828	positive regulation of glucose transport	1	5	0.030
GO:0008272	sulfate transport	1	5	0.030
GO:0048812	neuron projection morphogenesis	3	133	0.031
GO:0034622	cellular macromolecular complex assembly	4	234	0.032
GO:0008206	bile acid metabolic process	1	6	0.035
GO:0015129	lactate transmembrane transporter activity	1	6	0.035
GO:0016538	cyclin-dependent protein serine/threonine kinase regulator activity	1	6	0.035
GO:1903307	positive regulation of regulated secretory pathway	1	6	0.035
GO:0015727	lactate transport	1	6	0.035
GO:0030261	chromosome condensation	1	6	0.035
GO:0008271	secondary active sulfate transmembrane transporter activity	1	6	0.035
GO:0035873	lactate transmembrane transport	1	6	0.035
GO:0006027	glycosaminoglycan catabolic process	1	6	0.035
GO:0035493	SNARE complex assembly	1	6	0.035
GO:0035774	positive regulation of insulin secretion involved in cellular response to glucose stimulus	1	6	0.035
GO:0015116	sulfate transmembrane transporter activity	1	6	0.035
GO:0044421	extracellular region part	9	904	0.038
GO:0035082	axoneme assembly	1	7	0.040
GO:0045939	negative regulation of steroid metabolic process	1	7	0.040
GO:0017158	regulation of calcium ion-dependent exocytosis	1	7	0.040
GO:0032060	bleb assembly	1	7	0.040
GO:0043248	proteasome assembly	1	7	0.040
GO:0010894	negative regulation of steroid biosynthetic process	1	7	0.040
GO:0006270	DNA replication initiation	1	7	0.040

GO:0006026	aminoglycan catabolic process	1	7	0.040
GO:0005326	neurotransmitter transporter activity	1	7	0.040
GO:0005328	neurotransmitter:sodium symporter activity	1	7	0.040
GO:1901565	organonitrogen compound catabolic process	2	61	0.040
GO:0006928	movement of cell or subcellular component	5	371	0.041
GO:0046942	carboxylic acid transport	2	62	0.041
GO:0070062	extracellular exosome	7	636	0.042
GO:1903561	extracellular vesicle	7	639	0.043
GO:0043230	extracellular organelle	7	640	0.043
GO:0060384	innervation	1	8	0.045
GO:0016597	amino acid binding	1	8	0.045
GO:0090023	positive regulation of neutrophil chemotaxis	1	8	0.045
GO:0010827	regulation of glucose transport	1	8	0.045
GO:0031593	polyubiquitin binding	1	8	0.045
GO:0046324	regulation of glucose import	1	8	0.045
GO:1902624	positive regulation of neutrophil migration	1	8	0.045
GO:0008028	monocarboxylic acid transmembrane transporter activity	1	8	0.045
GO:0071624	positive regulation of granulocyte chemotaxis	1	8	0.045
GO:0031526	brush border membrane	1	8	0.045
GO:0015711	organic anion transport	2	66	0.046
GO:0042379	chemokine receptor binding	1	9	0.049
GO:0008045	motor neuron axon guidance	1	9	0.049
GO:0072348	sulfur compound transport	1	9	0.049
GO:0008009	chemokine activity	1	9	0.049
GO:0005838	proteasome regulatory particle	1	9	0.049
GO:0090022	regulation of neutrophil chemotaxis	1	9	0.049

GO:0032024	positive regulation of insulin secretion	1	9	0.049
GO:0006520	cellular amino acid metabolic process	2	69	0.050
